# CaCO_3_ as an Environmentally Friendly Renewable Material for Drug Delivery Systems: Uptake of HSA-CaCO_3_ Nanocrystals Conjugates in Cancer Cell Lines

**DOI:** 10.3390/ma12091481

**Published:** 2019-05-07

**Authors:** Viviana Vergaro, Isabella Pisano, Roberto Grisorio, Francesca Baldassarre, Rosanna Mallamaci, Antonella Santoro, Gian Paolo Suranna, Paride Papadia, Francesco Paolo Fanizzi, Giuseppe Ciccarella

**Affiliations:** 1 Dipartimento di Scienze e Tecnologie Biologiche e Ambientali, Università del Salento & UdR INSTM di Lecce, Campus Universitario, Via Monteroni, 73100 Lecce, Italy; francesca.baldassarre@unisalento.it; 2 CNR NANOTEC - Istituto di Nanotecnologia c/o Campus Ecotekne, Università del Salento, Via Monteroni, 73100 Lecce, Italy; roberto.grisorio@poliba.it (R.G.); gianpaolo.suranna@poliba.it (G.P.S.); 3 Dipartimento di Bioscienze, Biotecnologie e Biofarmaceutica, Università degli Studi di Bari «Aldo Moro», Via E. Orabona 4, I-70125 Bari, Italy; isabella.pisano@uniba.it (I.P.); rosanna.mallamaci@uniba.it (R.M.); 4 Dipartimento di Ingegneria Civile Ambientale, Del Territorio, Edile e di Chimica (DICATECh), Politecnico di Bari Via Orabona 4, 70125 Bari, Italy; 5 Institute of Biomembranes, Bioenergetics and Molecular Biotechnologies (IBIOM), CNR, Via Amendola 165/A, 70126 Bari, Italy; antonellasantoro1975@gmail.com; 6 Dipartimento di Scienze e Tecnologie Biologiche e Ambientali, Università del Salento, Via Monteroni, 73100 Lecce, Italy; paride.papadia@unisalento.it

**Keywords:** calcium carbonate nanocrystals, protein corona, cellular uptake, human cancer models

## Abstract

Chemical and biochemical functionalization of nanoparticles (NPs) can lead to an active cellular uptake enhancing their efficacy thanks to the targeted localization in tumors. In the present study calcium carbonate nano-crystals (CCNs), stabilized by an alcohol dehydration method, were successfully modified by grafting human serum albumin (HSA) on the surface to obtain a pure protein corona. Two types of CCNs were used: naked CaCO_3_ and the (3-aminopropyl)triethoxysilane (APTES) modified CaCO_3_-NH_2_. The HSA conjugation with naked CCN and amino-functionalized CCN (CCN-NH_2_) was established through the investigation of modification in size, zeta potential, and morphology by Transmission Electron Microscopy (TEM). The amount of HSA coating on the CCNs surface was assessed by spectrophotometry. Thermogravimetric analysis (TGA) and Differential scanning calorimetry (DSC) confirmed the grafting of APTES to the surface and successive adsorption of HSA. Furthermore, to evaluate the effect of protein complexation of CCNs on cellular behavior, bioavailability, and biological responses, three human model cancer cell lines, breast cancer (MCF7), cervical cancer (HeLa), and colon carcinoma (Caco-2) were selected to characterize the internalization kinetics, localization, and bio-interaction of the protein-enclosed CCNs. To monitor internalization of the various conjugates, chemical modification with fluorescein-isothiocyanate (FITC) was performed, and their stability over time was measured. Confocal microscopy was used to probe the uptake and confirm localization in the perinuclear region of the cancer cells. Flow cytometry assays confirmed that the bio-functionalization influence cellular uptake and the CCNs behavior depends on both cell line and surface features.

## 1. Introduction

Different type of materials on the nanoscale were recently used in cancer therapy, including gold nanoparticles for radio/photodynaminc therapy [1,2]; polymeric nanoparticles [3,4,5], micelles and liposomes for chemotherapy [6,7,8]; magnetic nanoparticles for hyperthermia [9,10,11]; quantum dots [12,13,14] and carbon nanotubes as theranostic agent [15,16].

Calcium carbonate is an inorganic and a polymorphic material which represents a ubiquitous raw material in nature: it is widespread and continuously replenished by means of natural cycles in rivers, lakes, and oceans or formed as minerals in animals, in the form of shells and skeletons, and in caves as stalactites and stalagmites. The use of environmentally friendly nanomaterials is an essential issue for biomedical application [17,18]. It is important to deeply investigate the possible interaction between nanomaterials with cells, tissue, and human organs. Experimental evidences, in vitro and in vivo, report that by pulmonary exposure, the nanomaterials localize in the lungs [19], evoking biological and toxicological side effect [20,21,22,23].

Calcium carbonate nanocrystals (CCNs) are appealing due to their applications in different fields since they are endowed with several attractive properties, such as a high surface area, a controllable pore size distribution, and a good biocompatibility [24,25]. These features make CCNs an attractive biomaterial for various biomedical applications, especially in drug delivery systems [26,27,28,29,30]. For these reasons, commercial CCNs are produced from natural sources which are widely available worldwide.

Many synthetic procedures were proposed in the last years, but all proposed methods are far from being challenge-free, requiring the use of surfactants or high temperature during the process [31,32,33]. Furthermore, it is well known that the synthetical methodology directly affects the CCNs crystalline phase affording one of calcite, vaterite, aragonite, and amorphous calcium carbonate. In our previous work, a green, fast, and straightforward spray drying technique was developed to synthesize CCNs in the calcite phase [34], suitable for drug delivery application [25]. 

It is commonly known that usually any nanoparticles with diameter <200 nm are retained in tumors because of the enhanced permeability and retention (EPR) effect [35,36,37,38], but in spite of extended investigations and developments in the field of cancer therapy, the use of nanoparticles in clinical applications is very limited. For instance, despite suitable features of CCNs as carrier systems [25,27,28,29,30,39], they are unstable in biological environments, hindering their applications e.g., in long-term drug delivery.

However, intracellular trafficking and biological activity are influenced by the functionalization of the nanoparticles surface. The literature reports different protocols of nanoparticles functionalization with polymers, PEG, chemical functionalization with peptide, polysaccharides or other biomolecules [40,41,42,43,44,45]. In principle, when exposed to a physiological environment, the NPs surface is coated by several biomolecules, mainly proteins, resulting in the formation of a “crown”, called protein corona. This corona can transform many of the physicochemical properties of NPs such as size, surface charge, surface composition, and functionality, therefore changing the molecular individuality of NPs [39,46,47]. These modifications modulate the particle–cell interactions and could conspicuously influence the efficacy of the delivery and finally, the therapeutic effects [48,49,50]. The protein corona has been reported to affect cellular interactions [51], cytokine expression [52], and protein function [53], further interfering with the regular metabolism and organ function [54]. For this reason, it is important to acquire an understanding of the many interactions occurring at the interface between NPs and the biological environment, to have a way to predict the fate of injected NPs. The adsorption of proteins on NPs was suggested as an essential toxicity mediation and its study is part of an ongoing efforts to establish the predictive tools needed for safety assessment. Another emerging important concept to obtain a “stealth effect”, is the requirement to mask NPs with proteins increasing systemic circulation time of NPs in the blood [55].

Therefore, with the aim of enhancing the CCNs bioavailability and therapeutic response, we performed a protein functionalization with Human Serum Albumin (HSA), which allows initial protection from recruitment of blood proteins and from the following macrophages phagocytosis. Albumin forms a pure protein corona around CCNs by simple incubation. 

To that end, we performed three steps: (i) synthesis of CNNs via an environmentally friendly spray drying process, followed by stabilization through an alcohol dehydration method, in order to avoid difficult to control factors affecting the nucleation process and subsequent crystal growth; (ii) CCNs functionalization by formation of a pure protein corona with Human Serum Albumin (HSA), allowing protection from recruitment of blood proteins and from the subsequent macrophages phagocytosis, followed by investigating physical changes in size, zeta potential, and morphology by TEM [56]; and (iii) internalization kinetics of the modified CNNs in three human cancer cell lines, breast cancer (MCF7), cervical cancer (HeLa), and colon carcinoma (Caco-2), performed by cytofluorimetric assay through fluorophore functionalization, together with cellular localization obtained via confocal microscopy.

The results stemming from these studies will contribute to the future use of CCNs for biomedical applications, and could be adapted towards understanding the mechanism through which other inorganic NPs coated with proteins cross the cellular membrane.

## 2. Materials and Methods 

### 2.1. Reagents

The chemicals used were: calcium chloride dehydrate 99.99% (CaCl_2_·2H_2_O, Sigma Aldrich, Darmstadt, Germany), sodium hydrogen carbonate (NaHCO_3_) (pro analysis, Merck, Germany), fetal bovine serum (FBS, Sigma Aldrich, Darmstadt, Germany), penicillin–streptomycin solution (Sigma Aldrich, Darmstadt, Germany), sodium pyruvate (Sigma Aldrich, Darmstadt, Germany), Dulbecco’s modified eagle’s medium (DMEM) (Sigma Aldrich, Darmstadt, Germany), phosphate buffered saline, Dulbecco A (PBS, Oxoid). (3-Aminopropyl)triethoxysilane (APTES) (Sigma, USA), Hoechst 33342 (Sigma Aldrich, Darmstadt, Germany), Triton X-100 (Sigma Aldrich, Darmstadt, Germany). Human Serum Albumin (HSA), Fluorescein isothiocyanate isomer I (FITC), Thiazolyl blue formazan (MTT formazan) were purchased from Sigma Aldrich (Germany) and used without further purification. 

### 2.2. CaCO_3_ Nanocrystals Synthesis and Bioconjugation 

#### 2.2.1. CaCO_3_ Nanocrystals Synthesis Procedure

CaCO_3_ nanocrystals were obtained by a literature method [2]. The process was performed on a Büchi Mini Spray Dryer B-290. Two aqueous solutions of NaHCO_3_ (250 mL, 0.125 M) and CaCl_2_ (250 mL, 0.062 M) were drawn by two peristaltic pumps and mixed by means of a T junction. The flow rates (5.2 mL/min and 4.5 mL/min, respectively) were set in order to ensure a 1.2:1 volume mixing ratio. The resulting solution was allowed to flow into a water cooled two-fluid nozzle and sprayed (inlet temperature 140 °C, aspirator 100%, spray gas flow length 50 mm). Upon spraying, a white dry powder was quickly formed. The powder was recovered by the collecting vessel and washed thrice with deionized water and centrifuged to separate CaCO_3_ from the soluble reaction products or unreacted reagents. Eventually, to facilitate the water removal and to prevent the crystallization process, the powder was washed with isopropyl alcohol before drying at 50 °C with a rotavapor.

#### 2.2.2. Amino-Functionalization of CaCO_3_ Nanocrystals

An amount of 0.150 g of CaCO_3_ powder was added to 6.25 mL toluene and 0.5 mL (3-aminopropyl)triethoxysilane (APTES). Then, the mixture was ultrasonic dispersed for 30 min and incubated under vigorous magnetic-stirring at 90 °C for 20 h. After stirring, the mixture was centrifuged three times with toluene, at 13,200 rpm for 10 min, to separate the amino-modified CaCO_3_ nanocrystals from the reaction medium. Eventually, the modified CaCO_3_ nanocrystals was repeatedly washed with water, dried in vacuo and stored for subsequent use.

#### 2.2.3. Fluorescein Coupling of Amino-Functionalized CaCO_3_ Nanocrystals

Anamount of 20 mg of amino-functionalized CCNs was dispersed in 3 mL of distilled water and 5 mL of a FITC solution, prepared in absolute ethanol at a concentration of 0.3 mg/mL, and kept under stirring for 16 h at room temperature in a dark environment. After incubation, the obtained fluorescent nanocrystals were rinsed three times with ethanol and twice with deionized (DI) water to remove the physically adsorbed FITC molecules. Finally, the FITC-labeled CaCO_3_ nanocrystals were repeatedly washed with DI water, dried under vacuum and kept for application. The FITC labeled particles were used for fluorescence microscope observations and flow cytofluorimetry [57]. 

#### 2.2.4. Adsorption of HSA on CaCO_3_ nanocrystals

A weighted amount mass (25 mg) of CCNs (naked CaCO_3_, CaCO_3_-NH_2_, or CaCO_3_-NH_2_-FITC) was suspended in 5 mL of a 20 mg/mL solution of HSA in 10 mM phosphate buffer saline (PBS) solution (pH = 7.4 and 0.15 M NaCl) and left under stirring for 1 h at 37 °C. The powder was centrifuged, washed with PBS for three times and dried under vacuum at room temperature overnight. The HSA-loaded materials were characterized by DSC and light scattering.

### 2.3. Material Characterization

TEM images were collected with JEOL JEM 1400 with a LaB6 source at 120 kV for naked CaCO_3_, and CaCO_3_-NH_2_, and 80 kV for HSA-modified CCNs. The zeta potential and the hydrodynamic diameter of the nanoparticles were measured with a Malvern Zetasizer Nano ZS. 

For fluorescent samples UV–Vis absorption spectra were obtained on a Varian-Cary 500 spectrophotometer (Agilent, Santa Clara, CA, USA). The excited fluorescence spectra measurements were performed using a Varian Cary Eclipse spectrofluorimeter. Absorption and fluorescence spectra were measured in DI water, with a concentration of 0.1 mg/mL. The quartz cuvettes used were of 1 cm path length.

In order to study cellular localization Zeiss LSM 700 confocal laser scanning microscope (Zeiss, Germany) was used. A 64× oil-immersion objective was used for the fluorescence measurements. The fluorescent samples were excited with a 0.5 mW laser of λ = 488 nm. The fluorescence intensity of samples was calculated from the microscopic images by the software of Zen 2009.

#### 2.3.1. Fluorescence Stability of FITC-Coupled Nanocrystals

Procedure (1): stability in physiological solution (PS). FITC-coupled nanoparticles (1.0 mg/mL) were dispersed in PBS (pH = 7.4). The samples were incubated at 37 °C under dark. At fixed time intervals, a 0.5 mL amount of solution with the suspended nanoparticles was sampled. The supernatant solution was collected after centrifugation to analyze the amount of cleaved FITC by UV−vis (λem=493 nm). To avoid the effect of FITC protonation state on the extinction coefficient, the supernatant was diluted 1:5 with PBS before the measurement. The quantification of FITC detachment was based on the Beer−Lambert law in which the ε of FITC in PS was 80,300 M^−1^·cm^−1^. 

Procedure (2): photostability tests. Photobleaching experiments were also conducted with a Zeiss LSM 700 microscope. FITC-coupled CCNs (1.0 mg/mL) were dispersed in HEPES buffer solution (4-(2-hydroxyethyl)-1-piperazineethanesulfonic acid). A selected area was excited continuously with a 0.5 mW/488 nm laser. The intensity of the fluorescence was simultaneously recorded at 2.0 s intervals by the fluorescence microscope system. As the control sample, the corresponding photobleaching test of pure FITC in HEPES was also carried out with the same procedure. The measurement of each sample was repeated three times with the same parameters. To present the results with standard error marks more illustratively, the results of fluorescent intensity after bleaching for 5, 10, 15, 20, and 30 min were adopted.

#### 2.3.2. Evaluation of HSA Adsorbed on CaCO_3_ Nanocrystals 

After the adsorption protocol described in the previous section, nanocrystals powders were washed with DI water for three times. The residual concentration of HSA in the supernatant was determined spectrophotometrically (λ = 562 nm) by using the bicinchoninic acid (BCA) assay. This method, based on the formation of a violet complex between Cu^+^ ions and the protein, is highly sensitive and suitable for the determination of a wide range of different proteins [58]. The amount of HSA adsorbed was calculated as a difference from the starting concentration. The results are reported as the mean value of three replicates, assuming the standard deviation as evaluation of the error.

#### 2.3.3. TGA/DSC Measurements

Thermogravimetric analyses (TGA) and differential scanning calorimetry (DSC) measurements were carried out on a Q600 apparatus (TA Instruments, New Castle, DE, US). The measurements were performed under a nitrogen flow (100 mL·min^−1^) with a heating rate of 10 °C/min [59,60].

### 2.4. Internalization Assay

#### 2.4.1. Cell Culture

Human breast cancer (MCF7), colon carcinoma cell line (Caco-2) and cervical cancer cell lines (HeLa) were cultured in Dulbecco’s modified eagle medium (DMEM; Sigma Aldrich, Darmstadt, Germany) supplemented with 10% fetal bovine serum (FBS; Sigma Aldrich, Darmstadt, Germany), 1% glutamine, and 1% penicillin/streptomycin (Invitrogen, Carlsbad, California, USA) in a humidified incubator at 37 °C and 5% CO_2_ and 95% relative humidity.

#### 2.4.2. Cell Proliferation Assay

MCF7, Caco-2 and Hela cells were treated with naked CCNs, CCNs-NH_2_-HSA and CCNs-HSA at a concentration of 100 µg mL^−1^ for 24 h. Cell proliferation was evaluated by MTT assay, which measures the conversion of a tetrazolium compound into formazan by a mitochondrial dehydrogenase enzyme in live cells. Briefly, 15 μL of MTT (3-(4,5-dimethylthiazol-2-yl)-2,5-diphenyltetrazolium bromide) was added to each well for 3 h at 37 °C. A solubilizing solution was added for 1 h, and the absorbance was then measured at 570 nm using a spectrophotometer. Each data point is the average of three independent determinations.

#### 2.4.3. Flow Cytometry 

Uptake kinetics. All cell lines at 2 × 10^5^ cells were seeded in 6-well plates (2 mL per well) in DMEM with 10% FBS and cultured overnight. The following day, cells were washed twice with PBS (1 mL) and kept in DMEM without FBS for 2 h. Then, 100 µg of CCNs diluted in culture medium were incubated with cells for different times, from 0.5 to 24 h at 37 °C and 5% CO_2_. After the incubation period, cells were washed twice with PBS, detached with 0.08% w/v trypsin, washed twice again in order to perform analysis on an Attune Acoustic Focusing Cytometer (Thermo Fisher Scientific) equipped with a 488 nm laser. The BL1 (530/30nm) detector was used for detection of green fluorescence from FITC.

ROS production. All cell lines at 2 × 10^5^ cells were seeded in 6-well plates (2 mL per well) in DMEM with 10% FBS and cultured overnight. The following day, cells were washed twice with PBS (1 mL) and kept in DMEM without FBS for 2 h. Then, 100 µg of CCNs diluted in culture medium were incubated with cells for 24 h at 37 °C and 5% CO_2_. After incubation period, cells were washed twice with PBS, and stained with a 10 µM dichorofluorescein (DCFA-DA) solution for 30 min and analyzed on a flow cytometer. As control cells were stained with propidium iodide (PI) 46 µM and a BL2 (575/24 nm) detector was used for detection of red fluorescence.

Confocal Microscopy. All cell lines were seeded on round coverslips at 6 × 10^4^ cells per slide one day prior to the assay. Particles were diluted prior to the assay as described for flow cytometry. Cells were washed with PBS, and 100 µg of CCNs, diluted in culture medium, were incubated with cells for 6 h at 37 °C and 5% CO_2_. 

After incubation, cells were washed with PBS, fixed in 0.25% glutaraldehyde in PBS for 10 min, and permeabilized with Triton X-100 (0.1% in PBS). The cells were then washed with PBS solution for 5 min and subsequently stained with 1 μg/mL of Hoechst 33342 for 5 min at room temperature.

Imaging tests were carried out with a Zeiss LSM700 (Zeiss, Germany) confocal microscope equipped with an Axio Observer Z1 (Zeiss, Goettingen, Germany) inverted microscope using a suitable oil-immersion objective (63X magnification, with 1.46 NA). Laser beams at λ = 405 nm and λ = 488 nm excitation wavelengths were used for imaging the nuclei and the fluorescent nanocrystals, respectively.

## 3. Results

### 3.1. Characterization of CaCO_3_ Nanocrystals

**Scheme 1:**
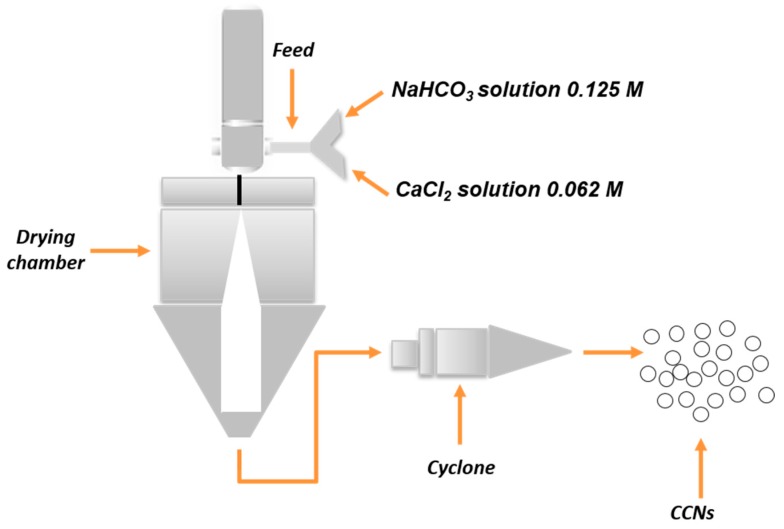
Synthesis of calcium carbonate nano-crystals (CCNs) by spray drying method.

The synthetic procedure of the CCNs has been previously reported in [25]. In Scheme 1 the spray-drying method applied is depicted.

**Scheme 2:**
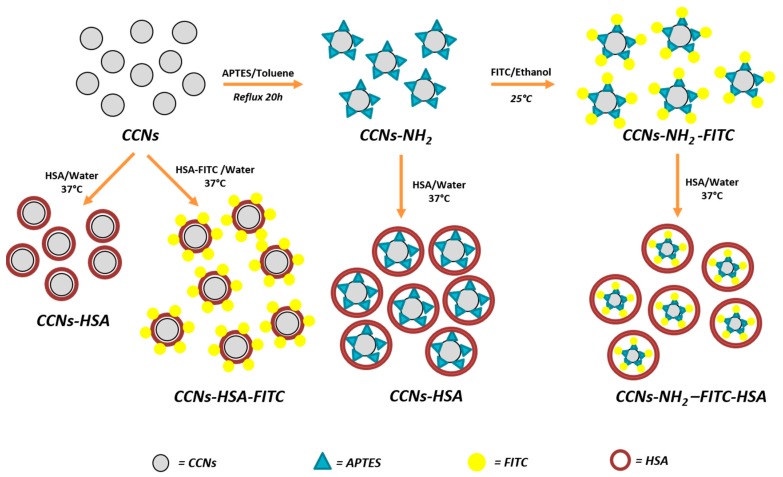
Representation of the main steps involved in the human serum albumin (HSA) functionalization of CCNs.

A detailed characterization of CCNs is reported in the supplementary Figure S1. It includes the morphological characterization performed by transmission electron microscopy, the physical–chemical characterization carried out by means of X-ray diffraction and Raman spectroscopy, as well as the surface charge and particles size assessment performed by Dynamic Light Scattering. Scheme 2 reports the steps involved in the chemical and bio-functionalization of the CCNs. The reaction with (3-aminopropyl)triethoxysilane (APTES) introduces the amino functional group on the CCNs surface. This group is necessary for the next functionalization step with fluorescein-isothiocyanate (FITC) in order to promote covalent grafting. 

The calculated mean particle diameter and zeta potential values are summarized in Table 1. Naked-CCNs show a higher hydrodynamic diameter (d_H_), probably due to the aggregation phenomena occurring in water. This observation is also confirmed by the lowest (−12.5 mV) zeta potential value. As expected and as already reported in the literature [61], the amino modifications improve the colloidal stability (Poly dispersion index (pdI) = 0.313) and increase the surface charge to a positive value (+14.8 mV). After interaction with HSA, the CCNs are surrounded by a protein cloud. The CCNs-HSA and CCNs-NH_2_-HSA complexes appeared to be smaller in terms of hydrodynamic diameter. The average diameters of the complexes ranged from 400 to 500 nm and from 200 to 300 nm, respectively. A significant modification of the zeta potential values occurs: from −12.5 ± 1.23 to −19.4 ± 1.77 for the pristine CCNs, and from +14.8 ± 0.693 to −24.9 ± 0.618 for the amino-functionalized sample. In general, the biological interaction between NPs and proteins depends on different physical forces: van der Waals interactions, hydrogen bonding, and electrostatic and hydrophobic interactions [23]. 

Similar results were obtained with mesoporous silica nanoparticles [62] where Nairi and co-workers underlined that surface charge was influenced by its functionalization; in particular, silica nanoparticles showed a negative zeta potential and this value, after amino-functionalization, became strongly positive. Likewise, the biopolymer functionalization, performed with bovine serum albumin, led to a decrease of zeta potential value. They also demonstrated that the bio-interaction occurs both with negative and positive charged nanoparticles, confirming that in biological systems non-electrostatic van der Waals interactions exist. 

**Figure 1:**
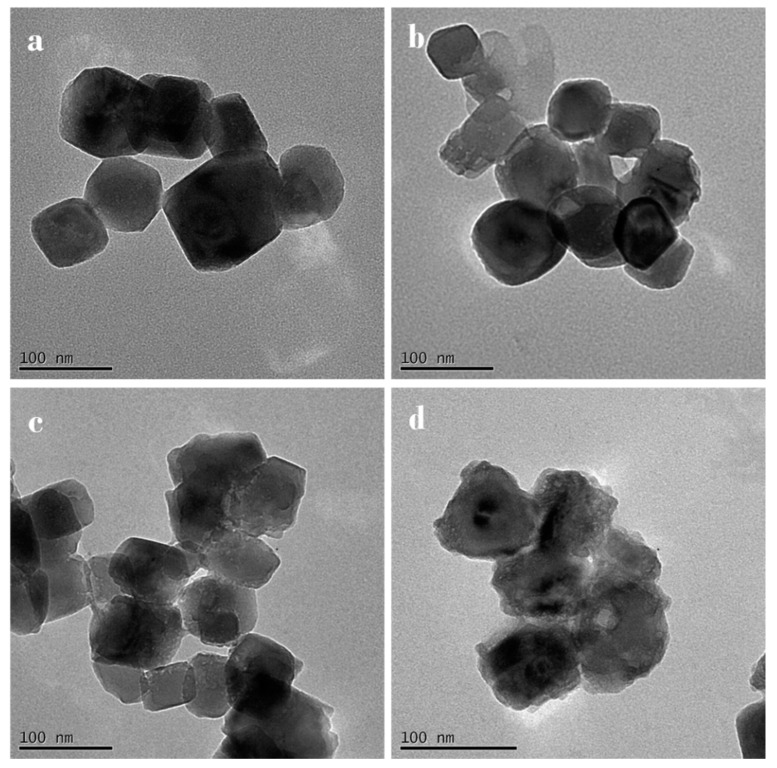
TEM characterization: (**a**) CCNs, (**b**) CCNs-HSA, (**c**) CCNs-NH_2,_ and (**d**) CCNs-NH_2_-HSA. Scale bar = 100 nm.

Based on this comparison, a TEM characterization was performed to verify the presence of the protein cloud surrounding CCNs and CCNs-NH_2_ nano-crystals. Figure 1 shows the TEM images of monodispersed nano-crystals before (Figure 1a,c) and after bio-conjugation (Figure 1b,d). After image analysis the measured diameter was found to be around 50–70 nm both for naked CCNs and CCN-NH_2_. Bioconjugated nano-crystals show a larger diameter: CCNs-HSA diameter ranged from 60 to 100 nm, while CCN-NH_2_-HSA show a diameter from 80 to 110 nm. For the last sample the presence of a globular protein around the surface is more visible (Figure 1d). 

The measurement of the protein interacting with the particles was calculated using the BCA assay. The amount of protein adsorbed on the CCNs surface differs significantly for the considered samples. In particular, we observed a larger amount of protein in the amino-functionalized samples (75.8% bio-conjugation efficiency) with respect to the pristine CCNs (43.2% bioconjugation efficiency). These data suggest that HSA has a higher affinity for the CCN-NH_2_ surface with respect to naked CCNs. Also Xu et al., demonstrated that the amino-functionalization of NPs could influence also the biofunctionalization [61].

We justified this behavior both for the strong negative charge of the HSA molecule, which can better interact with positive amino-functionalized CCNs-NH_2_ surface, and with the colloidal stability of nanocrystals: CCN-NH_2_ samples are smaller than the other ones and are more stable in water as shown before in the Dynamic Light Scattering (DLS) characterization. Probably, the aggregation occurring between naked CCNs reduced the active sites available for the interaction with HSA. These data suggest a complex interaction between the HSA protein and the calcium carbonate surface depending on different factors including the nature of both protein and solid surface as well as the surrounding environment.

### 3.2. TGA–DSC Measurements

**Figure 2:**
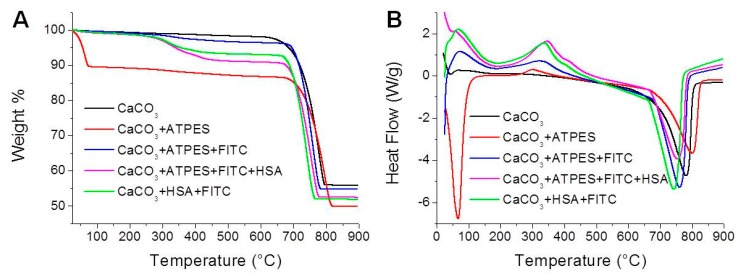
Thermogravimetric Analyses (**A**) and Differential Scanning Calorimetry (**B**) characterization of the synthesized nanoparticles.

In order to ascertain the adsorption of ligands onto the inorganic nanoparticles [63], thermogravimetric analyses (TGA) have been carried out on the suitably modified CaCO_3_ nanocrystals (Figure 2A). The TGA profiles of these samples evidence a similar behavior at high temperatures (600÷850 °C) as a consequence of the weight loss during the decomposition of calcium carbonate (CaCO_3_ → CaO + CO_2_). While neat nano-CaCO_3_ exhibits only the above-mentioned decomposition event, the other samples contain one or more phases with weight losses clearly evident at lower temperatures (< 600 °C) and therefore ascribable to the decomposition of the ligands surrounding the inorganic core. Furthermore, CCN–NH_2_ also exhibited the loss of residual solvent, such as dH_2_O derived from functionalization procedure. More insight into the decomposition of the organic components in these samples can be obtained by Differential Scanning Calorimetry (DSC) measurements (Figure 2B). The thermal event associated with decomposition of ATPES occurs at ~304 °C in CCN–NH_2_, which is remarkably higher than the boiling point (217 °C) of the compound. This observation strongly suggests the covalent grafting of APTES at the nanocrystal surface [64], which effectively hampers the volatilization of the molecule. The FITC adsorption was confirmed by the shift of the onset of the thermal decomposition of the organic shell at ~338 °C in the case of CCNs-NH_2_-FITC sample. Additional thermal events at ~410 °C confirmed the adsorption of HSA in the two samples containing the protein. It can be concluded that the thermal investigations on the synthesized nanoparticles confirmed the adsorption of ligands and in some cases the grafting onto the inorganic substrate.

### 3.3. Characterization of FITC-Coupled Nanocrystals

#### 3.3.1. Coupling Efficiency

In order to investigate the uptake and localization of nanocrystals inside cancer cells, confocal laser scanning microscopy (CLSM) and FACS analysis were performed using fluorescent nanocrystals. Green fluorescent CCNs were synthesized using a facile two step chemical process. In the first step the amino groups were introduced by silanization using (3-aminopropyl)triethoxysilane (APTES), and in the second one a covalent bonding was obtained between fluorescein isothiocyanate (FITC) groups and the amino group exposed on the CCNs surface. After functionalization, a dialysis step was performed, to ensure that no free fluorophore was present in the nanocrystal suspensions. This point is crucial both for the CLSM experiment as well as for the FACS measurements. To confirm this, the fluorescence related to CCNs was measured before and after dialysis.

**Figure 3:**
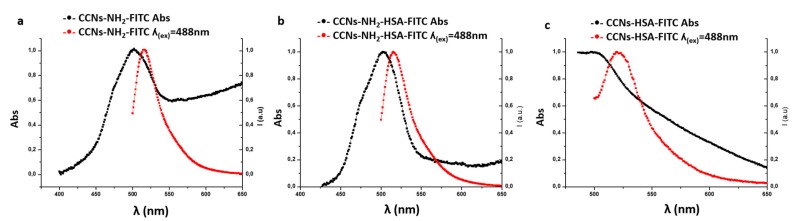
Absorbance and emission spectra of CCNs-NH_2_-FITC (**a**), CCNs-NH_2_-FITC-HSA (**b**) and CCNs-HSA-FITC (**c**).

The efficiency of FITC coupled on amine-modified CCNs was performed by measuring the absorbance and the emission spectra of functionalized nano-crystals by a spectrofluorimeter (Figure 3a).

As already reported in the literature [65,66], the vertical polymerization phenomenon occurs because of the chemical property of the aminosilane molecules, which form amine multilayers. The presence of the amine group on the CCNs surface, introduces a higher steric hindrance. Consequently, CCNs-NH_2_ samples showed higher FITC coupling efficiency due to the availability of surface amino groups. This happens because the isothiocyanate groups specifically react under bland conditions with the amino groups on the CCNs-NH_2_ surface, whereas no reaction occurs under the same conditions, with the naked surface of CCNs (data no shown). After this chemical synthesis reaction, the fluorescence from the CCNs became clearly visible (Figure 3a). Furthermore, the APTES modified samples are more suitable for further bio-functionalization. 

Following this functionalization, the bio-conjugation with HSA was performed in the dark and the optical properties of the relevant nanocrystals were studied by recording their absorbance and fluorescence spectra (Figure 3b). A third sample was used as reference: it was prepared using a commercially available HSA-FITC coupling (Sigma Aldrich). In this case the bio-functionalization was performed on naked CCNs (Figure 3c). The spectra of the latter are reported in Figure 3c. 

#### 3.3.2. Fluorescence Stability

**Figure 4:**
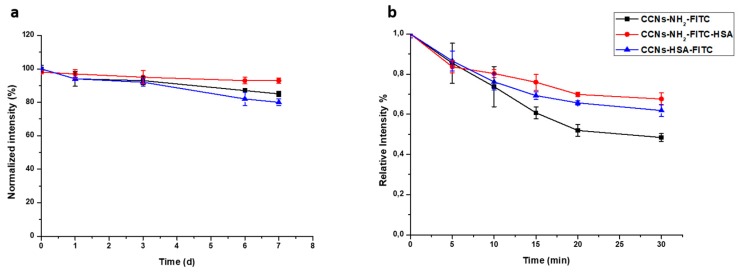
(**a**) Fluorescence emission stability of (■ black) CCNs−NH_2_-FITC (● red) CCNs−NH_2_-FITC-HSA, (▲ blue) CCNs-HSA-FITC in PBS at 37 °C. The error bars report the standard deviation of the measurements (three replicates). (**b**) The fluorescence intensity in MCF-7 cells was determined by staining with FITC-coupled samples by confocal microscopy. The fluorescence intensity evolution in the consecutive t-scan mode (λ_em_ = 488 nm) was monitored at 2 min intervals for a total of 30 min. Data were obtained from replicate (n = 5) experiments.

To assess the stability of fluorescent functionalization, FITC-conjugated samples were incubated in phosphate buffer solution at 37 °C in dark conditions, and the fluorescence signal was estimated at different time points for one week. Before the UV−vis spectrometer measurements, the samples were washed by centrifugation and rinsed twice with DI water. The time evolution of the normalized fluorescence intensity is reported in Figure 4a. The results showed that CCNs-NH_2_-FITC-HSA synthesized samples exhibited a higher fluorescence stability with respect to the CCNs-NH_2_-FITC and CCNs-HSA-FITC after the incubation in PBS for 7 days. In fact, these latter two exhibited a decrease in fluorescence intensity over time not lower than 10.0%. 

In the case of CCN-NH_2_-FITC, the result could be explained by invoking the progressive hydrolysis of the surface amine in PBS: under these experimental conditions an amount of FITC molecules detaches from the fluorescent amino-functionalized nano-crystals. It can be assumed that, after bio-conjugation with HSA the fluorescence is very stable because the protein steric hindrance prevents fluorophore detachment. In the case of the CCNs-HSA-FITC sample, the detachment of FITC likely occurs because the fluorophore is directly exposed to the solvent thereby promoting its release from the nanocrystals. 

Moreover, for biomedical application it is crucial considering the photo-stability of FITC-conjugated nanocrystals in a biological environment, because it is usually suitable to observe the NPs for a long time (Figure 4b). Photosensitivity is one of the principal matters for fluorescence labeling because, in biological environments, the fluorescent molecules undergo photo-oxidation, which lead them to lose their photoluminescent property.

For this purpose, the photo-stability of FITC-coupled samples was studied by confocal microscopy, in particular, FITC-coupled samples were incubated with MCF7 cells, in complete culture medium, for 2 h and immediately imaged by CLSM. During imaging, the living cells were continuously irradiated by 0.5 mW laser beam at λ = 488 nm, and the green fluorescence intensity was measured by software over time. 

Then the green fluorescence intensity of the internalized fluorescent CCNs was normalized and plotted as a function of time. The results are reported in Figure 4b. The CCN-NH_2_-FITC-HSA exhibited a slow fluorescence intensity decrease in the course of five scans for a total irradiation time of 30 min. As expected, CCN-NH_2_-FITC exhibited a very fast photobleaching behavior: the relative intensity of fluorescence was reduced by 50% within 20 min. The weak fluctuations of the fluorescent intensity may be due to the metabolic activity of the living cells. These data demonstrated a higher photostability of the FITC-conjugated samples obtained by the proposed two step chemical reactions.

However, CCNs-HSA-FITC showed a moderate photostability. After constant laser excitation, these FITC-coupled nanocrystals samples suffer only poor photobleaching. The reason was probably due to the chemical bound with HSA which reduced the rotational and vibrational degrees of freedom as compared to free molecules. This bonding increases the photostability of the sample [67].

### 3.4. Internalization Assay

**Figure 5:**
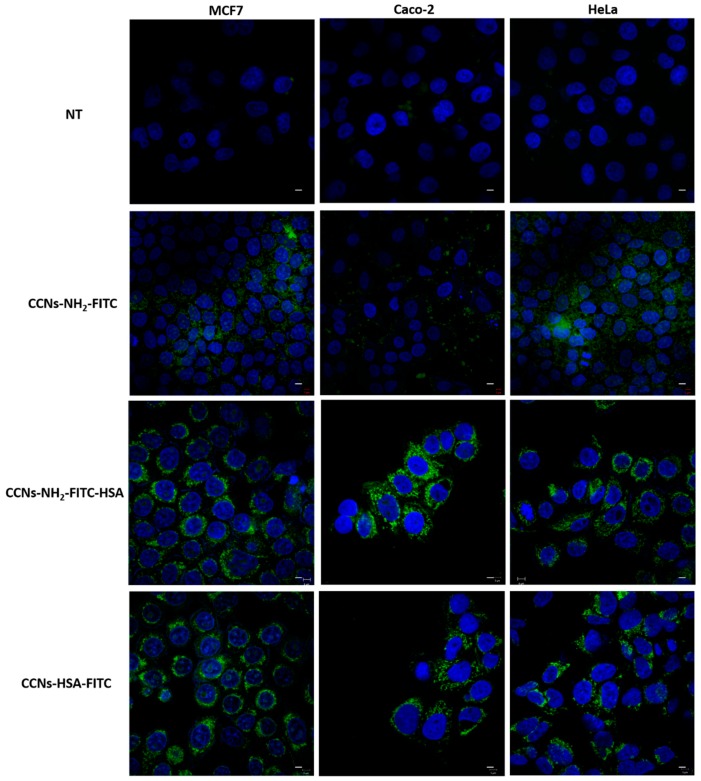
Confocal microscopy images evidencing cellular uptake of CCNs-FITC samples by MCF-7, HeLa and Caco-2 cells. The nucleus was stained by using Hoechst 33342 (blue) after cells fixation. A FITC filter responding to green fluorescence signals was employed to recognize the location of FITC conjugated CCNs: CCN-NH_2_-FITC, CCNs-NH_2_-FITC HSA and CCNs-HSA-FITC. Scale bar 5 µm.

The cell internalization of CCN-NH_2_-FITC, CCNs−NH_2_-FITC-HSA and CCNs-HSA-FITC samples was probed by confocal microscopy. For this purpose, MCF-7, HeLa, and Caco-2 cells treated in complete culture medium for 2 h with the same concentration of CCNs-FITC sample (100 µg/mL) were analyzed. To assess the presence of the CCNs in each sample, the nucleus was stained using Hoechst 33342 (blue) after the cell fixation process. A FITC filter recognizing the green fluorescence signals was employed to visualize the CCNs-FITC sample. The first line of Figure 5 shows confocal microscopy images of non-treated samples to verify the presence of cell autofluorescence. The second line imaged the internalization of CCN-NH_2_-FITC. In this case it can be seen how the fluorescence is diffused throughout the cytoplasm. In addition, in MCF-7 and HeLa cells the entire cytoplasm appeared as uniformly green-colored, suggesting a diffuse localization within the cytoplasm. 

**Figure 6:**
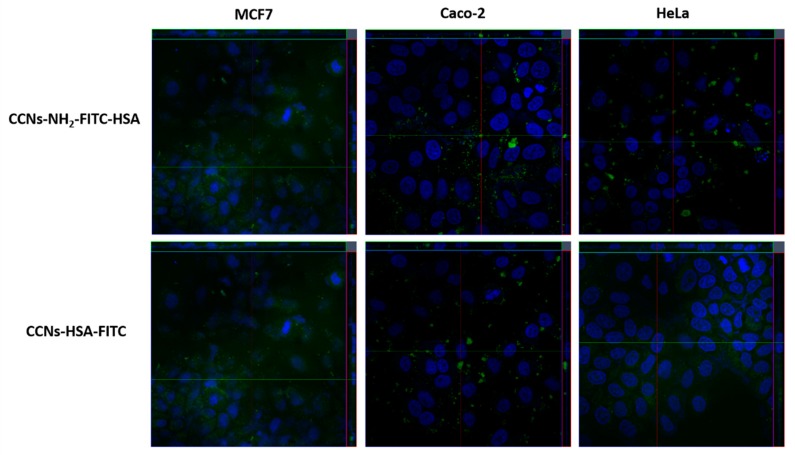
Z-Stack confocal microscopy images showing cellular uptake of CCNs-NH_2_-FITC HSA (first line) and CCNs-HSA-FITC (second line) by MCF7, HeLa and Caco-2 cells. The nucleus was stained by using Hoechst 33342 (blue) after cells fixation. A FITC filter responding to the green fluorescence employed to recognize the location of FITC conjugated CCNs.

Conversely, for cells exposed to CCNs−NH_2_-FITC-HSA (Figure 5, third line) and CCNs-HSA-FITC (Figure 5, bottom line), a significant increase in green fluorescence intensity was evident in the perinuclear region of the cancer cells. To confirm these data, the CCNs internalization, the z-stack process was recorded. Figure 6 shows a z-stack section for CCNs−NH_2_-FITC-HSA and CCNs-HSA-FITC. Confocal microscopy investigation demonstrated that all samples were rapidly and efficiently uptaken by cancer cells.

**Figure 7:**
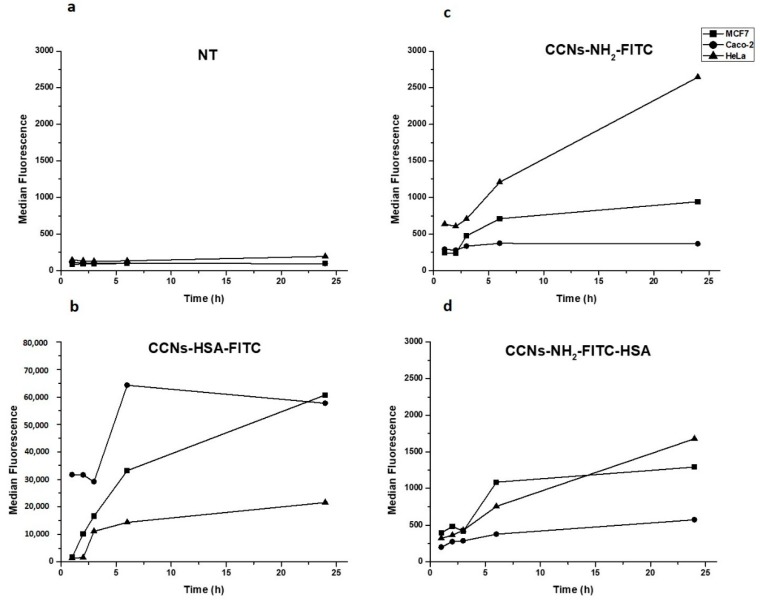
Uptake kinetics of non-treated sample NT (**a**), CCNs-NH_2_-FITC (**b**), CCNs-NH_2_-FITC-HSA (**c**) and CCNs-HSA-FITC (**d**) performed in MCF7 (■), Caco-2 (●) and HeLa (▲) cells. Data were obtained from replicate experiments (n = 3).

Based on the above results, we carried out further measurements performing a flow cytometry assay in order to understand in depth the correlation between the protein corona and the cellular uptake kinetics (Figure S2). Figure 7 shows the median fluorescence of each sample measured by cytofluorimetric assay: obviously, the samples tested were all FITC-labelled and the kinetics was assessed at fixed times, from 30 min up to 24 h. The kinetics profiles depend on the different cell lines and sample type. The controls (NT) evidenced that the cells autofluorescence is very low. 

In MCF7 cell line the synthesized nanocrystals, CCNs-NH_2_-FITC and CCNs-NH_2_-FITC-HSA, are uptaken with faster kinetics than the CCNs prepared by reaction with commercial HSA-FITC sample: the curves in (c) and (d) reached saturation faster just after 6 h of incubation, while in the case of the curve obtained in (b), under the same conditions, the fluorescence intensity barely reached 50% in comparison to the fluorescence intensity measured at 24 h. Considering the Caco-2 cell line the situation is the following: the curves (c) and (d) reached saturation after 1 h, while in the curve (b) an incubation time of 6 h is necessary. Finally, in the HeLa cell lines, the behavior of CCNs-NH_2_-FITC and CCNs-NH_2_-FITC-HSA seem to be time dependent because the fluorescence intensity increases during the incubation time, but the kinetics in (b) seems to reach linearity after 6 h. 

The data obtained in this study are in agreement with the literature; indeed, the kinetics depends on both the NPs stability, surface functionalization and the cell lines used. In general, the amino-functionalized NPs are rapidly internalized by cells [68]. Owing to the HSA protein on the CCNs surface, the uptake efficiency is higher, and the size-dependent uptake pattern is different from that of naked nanomaterials, as already demonstrated [69].

**Figure 8:**
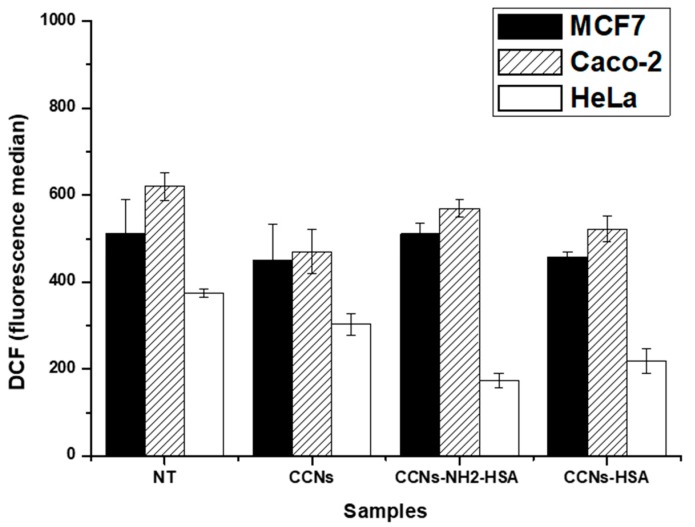
Reactive Oxygen Species (ROS) fluorescence intensity measured in MCF7 (black histogram), Caco-2 (patterned histogram) and HeLa (white histogram) cells. The experimental conditions are non-treated cells (NT), naked CCNs, CCNs-NH_2_-HSA and CCNs-HSA. Data were obtained from replicate experiments (n = 3).

Once demonstrated that the plasma proteins adsorbed onto the CCNs surface influence cellular uptake, we ascertained also if the exposition to nanomaterials influences the cell viability and Reactive Oxygen Species (ROS) production. To perform these experiments, no fluorescent samples were used. After 24 h of incubation with nanomaterials at a concentration of 100 µg mL^−1^, both naked and functionalized CCNs, had nearly no cytotoxic effects on MCF7, HeLa, and Caco-2 cell lines (PI and MTT assay Figures S3 and S4, respectively). Regarding oxidative stress production, a specific fluorescent agent was used to identify the cellular expression of ROS. Figure 8 shows the media measurements of ROS production: in all cell lines, both naked CCNs and HSA-functionalized CCNs, ROS levels are in the same rage of non-treated sample (NT histogram in Figure 8). It is important underline that Hela cells showed the lowest fluorescent signal. The reason for this behavior probably was due to the major stability of the cell line to oxidative stress in the presence of nanocrystals. 

In accordance with our results, Mo and co-workers reported that the presence of a protein corona around NPs led to a reduction of toxicity to cells [69,70]. Furthermore, in vitro and in vivo experiments, demonstrated that the presence of protein corona around Ag NPs influence the toxicity [71]. This is an important point for biomedical application, because a detailed investigation of the interaction between biofunctionalized NPs and cancer cells helps us to understand their impact on blood circulation timeas well as immunostimulotory and immunosuppressory effects [72,73,74,75]. 

## 4. Conclusions

In order to improve targeting efficiency and limit the systemic toxicity effects of CCNs, we studied methods to graft HSA on nanocrystals and obtain a pure protein corona. Two pathways were compared: a simpler, direct method (using unmodified environmentally friendly CaCO_3_), and a surface functionalization with amino groups (using (3-aminopropyl)triethoxysilane to modulate the surface behavior of the nanocrystals). DLS, TEM, and TGA-DSC analysis were used to confirm the functionalization of CCNs and study the conjugates. HSA coating reduced the hydrodynamic diameter and prevented aggregation or agglomeration of the nanocrystals. 

The amino groups proved essential to obtain the best bio-functionalization of CCNs with HSA, in comparison with pristine CCNs and HSA, as measured by the bicinchocinic assay. In fact, a larger amount of protein was observed in the amino-functionalized sample (75.8% bio-conjugation efficiency) with respect to the pristine CCNs (43.2% bio-conjugation efficiency). DSC confirmed the presence of a specific thermal event associated with the presence of HSA in the samples. Confocal microscopy on FITC-labeled samples confirmed that three human cancer cell lines MCF-7 (breast cancer), Caco-2 (epithelial colorectal adenocarcinoma), and HeLa (cervical cancer), efficiently internalize CCNs−NH_2_-FITC-HSA and CCNs-HSA-FITC. Moreover, while the fluorescence of non-HSA-functionalized samples was dispersed in the cytoplasm, z-stack sections confirmed the localization of the conjugates in the perinuclear region. 

These results suggest that the CCNs-HSA conjugates show an improved uptake ability in cancer cell lines with respect to naked CCNs. The internalization studies verify that the endocytosis patterns depend on chemical functionalization and bioconjugation. The surface charge and the bio-functionalization affect the efficiency and the pathway of cellular uptake. 

All these results indicate that CCNs-HSA conjugates deserve further insights as targeted nanocarriers of anticancer compounds.

## Figures and Tables

**Table 1:** Dynamic Light Scattering (DLS) characterization of CCNs samples.

Sample	Size (nm) (d_H_)	PDI Poly Dispersion Index	ζ-Potential (mV)
**CCNs**	800.1	0.501	−12.5 ± 1.23
**CCNs-NH_2_**	587.2	0.313	+14.8 ± 0.70
**CCNs-HSA**	497.2	0.467	−19.4 ± 1.80
**CCNs-NH_2_-HSA**	285.4	0.105	−24.9 ± 0.62

## Supplementary Materials

The following are available online at https://www.mdpi.com/1996-1944/12/9/1481/s1, Figure S1: CaCO_3_ physico-chemical characterization; Figure S2: Cytofluorimetric analysis: uptake kinetics; Figure S3: Cytofluorimetric analysis: Propidium Iodide assay; Figure S4: MTT assay.
