# Supplementary material for: CaCO3 as an Environmentally Friendly Renewable Material for Drug Delivery Systems: Uptake of HSA-CaCO3 Nanocrystals Conjugates in Cancer Cell Lines
Source: Materials (Basel). 2019 May 7;12(9):1481. doi: 10.3390/ma12091481 (PMC6539763; doi:10.3390/ma12091481)
Supplement: Supplementary file 1 [file materials-12-01481-s001.pdf]

Supporting Information

$CaCO_3$ as an environmentally friendly renewable material for drug delivery systems: uptake of HSA-$CaCO_3$ nanocrystals conjugates in cancer cell lines

Viviana Vergaro[1,2*], Isabella Pisano[3], Roberto Grisorio[2,4], Francesca Baldassarre[1,2], Rosanna Mallamaci[3], Antonella Santoro[5], Gian Paolo Suranna[2,4], Paride Papadia[6], Francesco Paolo Fanizzi[6*], Giuseppe Ciccarella[1,2*]

[1] Dipartimento di Scienze e Tecnologie Biologiche e Ambientali, Università del Salento & UdR INSTM di Lecce, Campus Universitario, Via Monteroni 73100 Lecce, Italia; viviana.vergaro@unisalento.it; francesca.baldassarre@unisalento.it; giuseppe.ciccarella@unisalento.it;

[2] CNR NANOTEC - Istituto di Nanotecnologia c/o Campus Ecotekne, Università del Salento, Via Monteroni 73100 Lecce, Italia; viviana.vergaro@unisalento.it; roberto.grisorio@poliba.it; francesca.baldassarre@unisalento.it; gianpaolo.suranna@poliba.it; giuseppe.ciccarella@unisalento.it;

[3] Dipartimento di Bioscienze, Biotecnologie e Biofarmaceutica, Università degli Studi di Bari «Aldo Moro», Via E. Orabona 4, Bari, I-70125, Italia; isabella.pisano@uniba.it; rosanna.mallamaci@uniba.it;

[4] Dipartimento di Ingegneria Civile Ambientale, Del Territorio, Edile e di Chimica (DICATECh), Politecnico di Bari Via Orabona 4, Bari, 70125, Italia; roberto.grisorio@poliba.it; gianpaolo.suranna@poliba.it

[5] Institute of Biomembranes, Bioenergetics and Molecular Biotechnologies (IBIOM), CNR, Via Amendola 165/A, 70126 Bari, Italy; antonellasantoro1975@gmail.com;

[6] Dipartimento di Scienze e Tecnologie Biologiche e Ambientali, Università del Salento, Via Monteroni 73100 Lecce, Italia; paride.papadia@unisalento.it; fp.fanizzi@unisalento.it.

* Correspondence: viviana.vergaro@unisalento.it; Tel.: +39-0832-319207; +39-0832-319810 (G.G.); (F.P.F.); +39-0832-319810 (G.G.)

Figure S1. $CaCO_3$ physico-chemical characterization

Figure S2. Cytofluorimetric analysis: uptake kinetics

Figure S3. Cytofluorimetric analysis: Propidium Iodide assay

Figure S4. MTT assay

a

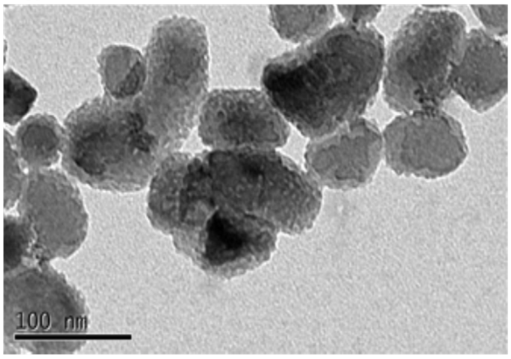

b

Size

Sample	Ultra-pure water	PBS	DMEM
	Mean size ± S.D(nm)/PDI	Mean Size ± S.D(nm)/PDI	Mean size ± S.D. (nm) /PDI
**$CaCO_3$NCs**	222.7±9.2/0.235	188.8±4.4/0.310	344.0±32.8/0.691

ζ-potential

pH	Ultra-pure water	PBS	DMEM
**7.4**	6.8±0.1	-25.1±0.3	-11.2±0.2
**5.5**	8.9±0.2	-28.4±0.1	-7.1±0.3

c

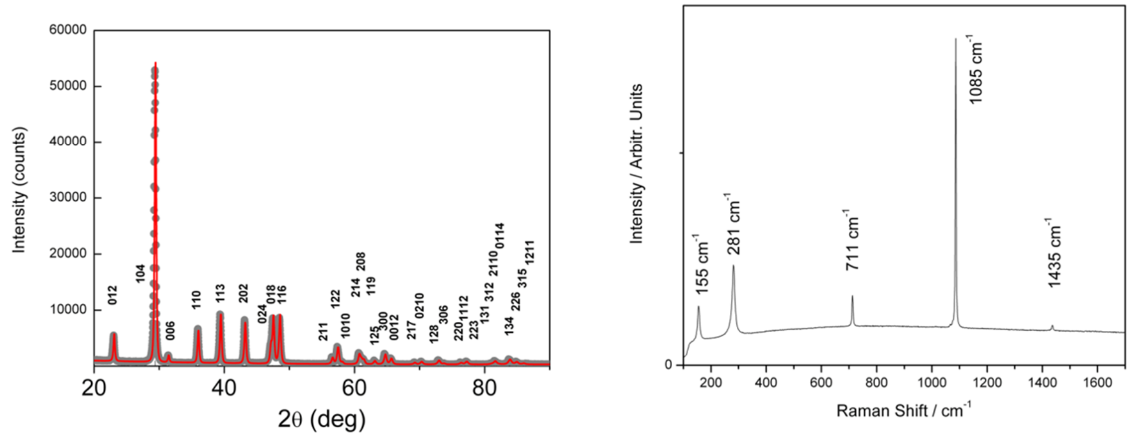

d

Figure S1. $CaCO_3$ chemical characterization: a) TEM image, scale bar 100 nm; b) Dynamic Light Scattering measurements (size and zeta-potential); c) XRD pattern; d) Raman spectrum.

The XRD pattern (Figure S1 c) was indexed as rhombohedral calcite phase (ICSD collection code 040544). The Raman spectrum (Figure S1 d) showed five bands which matched perfectly the literature one for calcium carbonate in calcite phase.

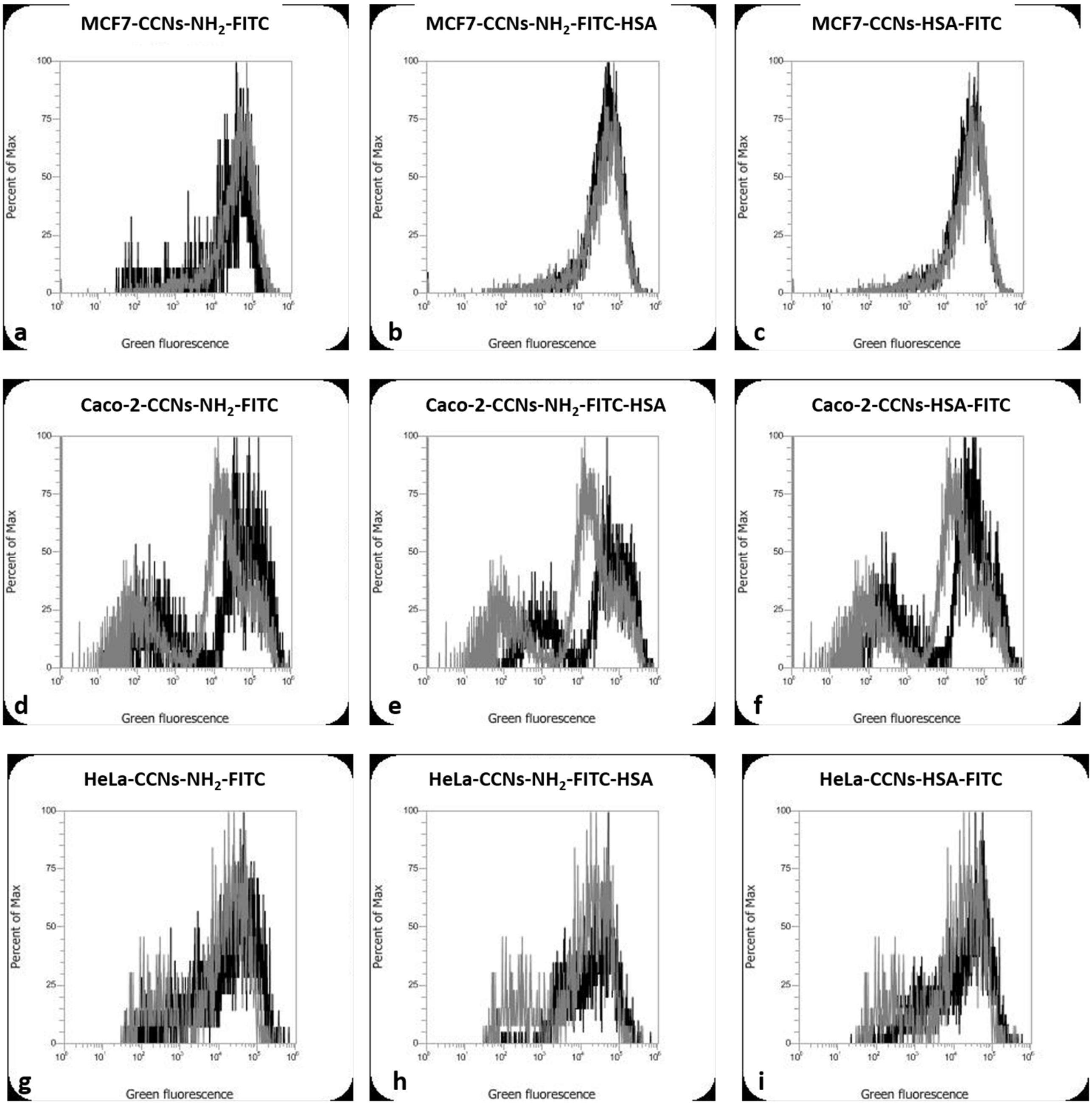

Figure S2. One representative flow cytometry measurements assessing the uptake kinetics of CCNs-NH_2-FITC (a, d, g), CCNs-NH_2-FITC HSA (b, e, h) and CCNs-HSA-FITC (c, f, i) performed in MCF7 (a-c), Caco-2 (d-f) and HeLa (g-i) cells.

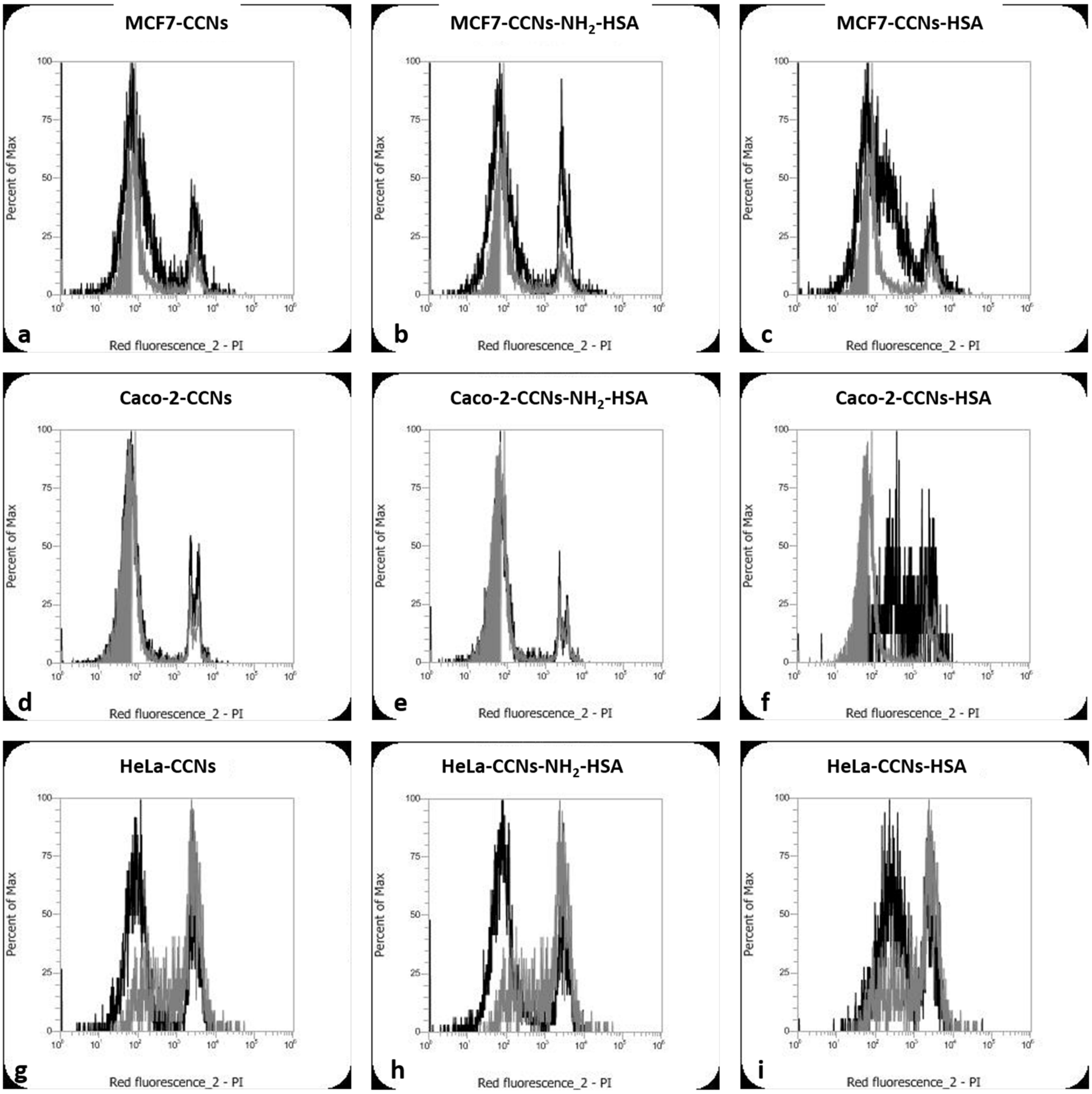

Figure S3. One representative flow cytometry measurements assessing the biocompatibility through Propidium Iodide assay of naked CCNs (a, d, g), CCNs-NH_2-HSA (b, e, h) and CCNs-HSA (c, f, i) performed in MCF7 (a-c), Caco-2 (d-f) and HeLa (g-i) cells.

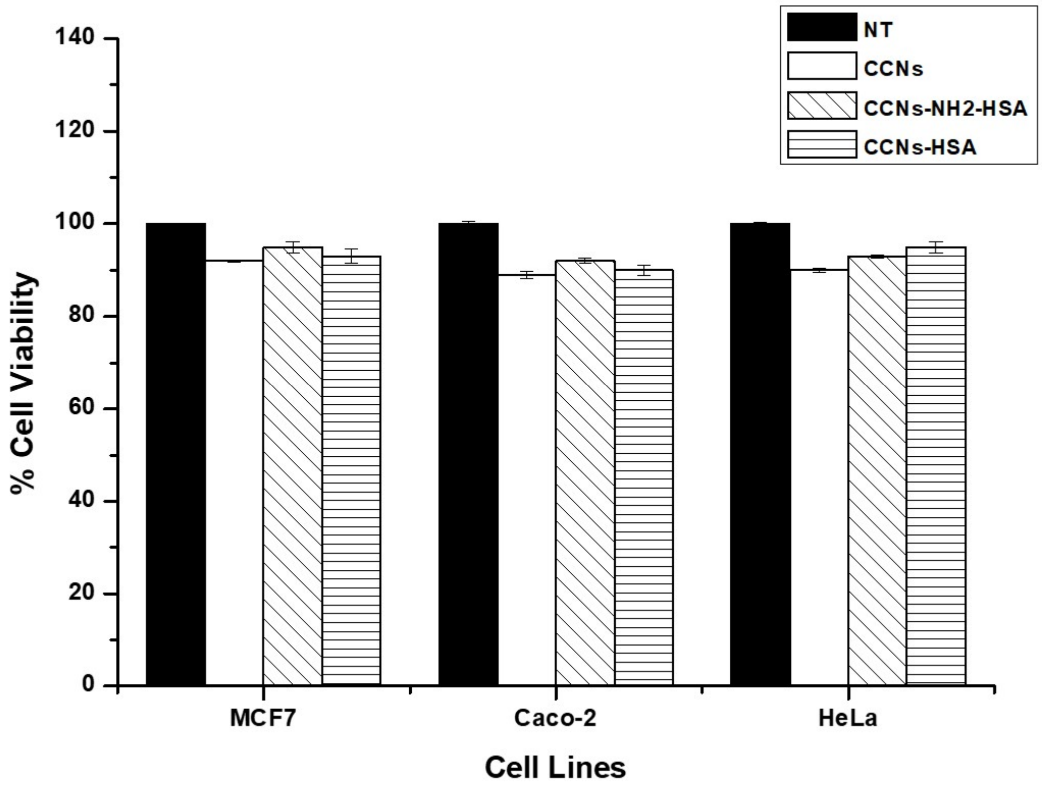

Figure S4. MTT assay to assess the biocompatibility of naked CCNs (white), CCNs-NH_2-HSA (diagonal lines) and CCNs-HSA (horizontal lines) performed on MCF7, Caco-2 and HeLa cells; the controls (NT = Non-treated cells) are labelled in black. Data were obtained from replicate experiments (n = 3).
